# STING Signaling Deficiency Exacerbates Demyelination and Immune Infiltration in Focal EAE Lesions

**DOI:** 10.3390/neurosci6040106

**Published:** 2025-10-17

**Authors:** Marlene T. Mørch, Line S. Reinert, Anouk Benmamar-Badel, Magdalena Dubik, Mark Burton, Mads Thomassen, Torben Kruse, Nasrin Asgari, Søren R. Paludan, Trevor Owens, Reza Khorooshi

**Affiliations:** 1 Department of Neurobiology, Institute of Molecular Medicine, University of Southern Denmark, Campusvej 55, 5230 Odense M, Denmark; 2 Department of Biomedicine, University of Aarhus, Høegh-Guldbergs Gade 10, 8000 Aarhus C, Denmark; 3 Aarhus Research Center for Innate Immunology, University of Aarhus, Høegh-Guldbergs Gade 10, 8000 Aarhus C, Denmark; 4 Department of Clinical Genetics, Odense University Hospital, J.B. Winslows Vej 4, Entrance 24, 5000 Odense, Denmark; 5 Department of Neurology, Slagelse Hospital, Institute of Regional Health Research, Fælledvej 13, 4200 Slagelse, Denmark

**Keywords:** STING, type I IFN, EAE, focal brain pathology

## Abstract

Stimulator of interferon genes (STING) is a cytosolic DNA sensor that activates type I interferon (IFN) signaling, which plays a key role in neuroinflammation. Although the role of STING in experimental autoimmune encephalomyelitis (EAE), a model of multiple sclerosis (MS), remains debated, its involvement in the development of CNS lesions, particularly within localized pathology, modeled here by targeting the corpus callosum, has yet to be explored. Using a focal EAE model, we compared the induction of lesions in wild-type and STING-deficient (STING^gt/gt^) mice. Lesions were analyzed by immunohistochemistry, flow cytometry, and transcriptomics. STING-deficient mice had significantly larger demyelinated lesions, reduced ISG expression, and modified immune cell infiltration. STING signaling limits lesion severity in focal EAE by promoting IFN responses and regulating immune infiltration. These findings position STING as a potential target for MS therapy.

## 1. Introduction

Pattern recognition receptors (PRRs) play a major part in the innate immune response. They recognize structures conserved among infectious agents as well as endogenous molecules associated with tissue damage. PRRs include transmembrane as well as cytoplasmic proteins, whose activation results in the increased transcription of genes including type I interferons (IFNs) [1,2]. Our group and others have shown that PRR signaling can play a role in modifying the disease course of experimental autoimmune encephalomyelitis (EAE), an animal model of multiple sclerosis (MS) [3,4,5] and prevent the formation of focal EAE lesions [6].

Stimulator of interferon genes (STING) is a critical adaptor protein in cytoplasmic DNA sensing pathways, playing a central role in innate immune responses. Upon activation by cyclic dinucleotides, STING triggers the transcription of type I IFNs and proinflammatory cytokines [2,7]. Type I IFNs are essential for antiviral defense, regulation of inflammation in the central nervous system (CNS), and homeostasis [8]. These cytokines signal through the IFN-α/β receptor (IFNAR), leading to the induction of interferon stimulated genes (ISGs) that regulate immune responses [8,9]. Among type I IFNs, IFN-β is clinically utilized as a disease-modifying treatment for MS [10]. Studies of EAE have demonstrated the upregulation of IFN-β during disease progression [11]. Mice lacking IFN-β or type I IFN signaling exhibit exacerbated disease severity [11,12].

STING has in recent years emerged as a key PRR signaling mediator, particularly in CNS inflammation [13]. STING protein expression has been shown to be altered in MS and EAE, with decreased expression in the periphery and increased expression in the CNS [14,15]. However, its role in EAE is debated, with some studies indicating that STING deficiency leads to a milder disease, others reporting that STING activation mitigates EAE, and some demonstrating no significant difference between STING-deficient and wild-type (WT) C57BL/6 mice [16,17,18]. Notably, STING-deficient mice exhibited overall altered EAE pathology, with differences in microgliosis and immune cell infiltration compared with WT controls [17]. However, specifically how STING signaling contributes to lesion development is unknown.

To further elucidate STING’s role in CNS pathology, particularly in lesion development, we utilized our focal EAE model [6]. Transcriptomic analysis of corpus callosum (CC) lesions revealed that nearly half of the upregulated genes were ISGs. Importantly, our investigation into PRR signaling pathways indicates that STING is a key regulator of lesion development. Mice lacking functional STING developed significantly larger lesions, characterized by altered monocyte and lymphocyte proportions, increased CD8+ T cells, and reduced ISG expression. These findings underscore the pivotal role of STING-mediated signaling in shaping neuroinflammatory responses and highlight its potential as a therapeutic target in MS and related disorders.

## 2. Materials and Methods

### 2.1. Mice

Adult female C57BL/6J mice were purchased from Taconic, Lille Skensved, Denmark and STING^gt/gt^ mice (C57BL/6J mutant mice lacking a functional STING protein, STING-deficient) [19] were bred at Taconic, Lille Skensved, Denmark. Mice were housed at The Biomedical Laboratory, University of Southern Denmark. All experiments were conducted in accordance with the National Ethical Committee, Animal Experiments Inspectorate under the Danish Ministry of Food, Agriculture, and Fisheries, and The Danish Veterinary and Food Administration (approval number 2020-15-0201-00652).

### 2.2. Focal EAE Lesion

**Figure 1:**
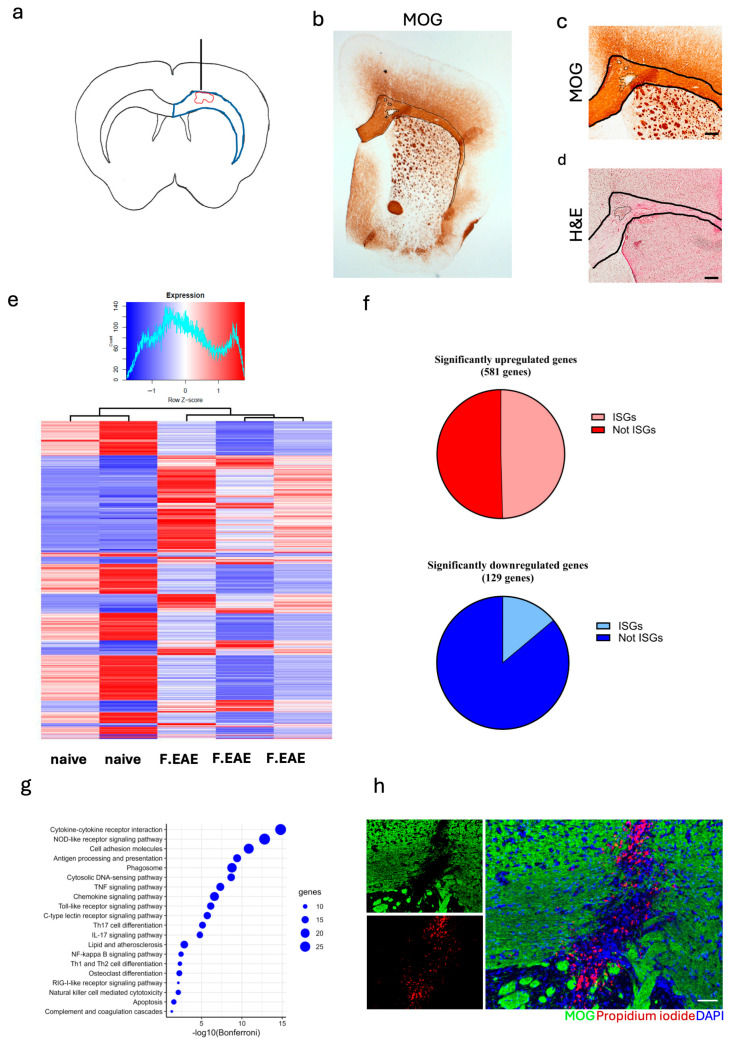
Type I interferon stimulated genes are heavily represented in focal lesions. C57BL/6 female mice were immunized with MOG 35-55 peptide and pertussis toxin on days 0 and 2. (**a**) Schematic overview of focal lesions induced by stereotactic insertion of a 30 G needle to corpus callosum 10 days after immunization, lesions were examined 16 days post immunization, blue line shows an outline of CC while the red line represents the lesion outline. (**b**) Representative overview micrograph of MOG staining showing lesion (dotted line) in the CC (solid line). (**c**) Representative micrograph of focal lesion in CC (solid lines) showing demyelination (dotted lines) identified using MOG immunohistochemistry. (**d**) Representative micrograph of focal lesion in CC (solid lines) showing classic infiltration (dotted line) identified with H&E staining. CC was micro-dissected from naïve/control and lesioned mice, and RNA was extracted for bulk RNA sequencing. (**e**) Heatmap of the 710 identified differentially expressed genes (LogFC ≥ ±1.5 and FDR ≤ 0.05) identified between the focal EAE lesioned and naïve CC; 581 were upregulated and 129 were downregulated. (**f**) Top pie chart showing the proportion (49.6%) of upregulated genes that were type I interferon stimulated genes (ISGs), bottom pie chart showing the proportions (14%) of downregulated genes that were ISGs. (**g**) Dot plot (ORA) representing the significant hits from the KEGG pathway enrichment analysis on the ISGs. (**h**) Representative micrograph of immunohistochemistry staining for MOG (green) and propidium iodine (red) staining showing cell-free DNA in the focal lesions in CC; propidium iodine was intrathecally injected into the cerebrospinal fluid 30 min prior to sacrifice. Scale bar for (**c**) + (**d**): 200 µm and for (**h**): 50 µm.

As previously described [6], mice (C57BL/6J and STING-deficient) were immunized by injecting 100 microliters (µL) of emulsion containing 100 µg MOGp35-55 (MEVGWYRSPFSRVVHLYRNGK, TAG Copenhagen A/S, Frederiksberg, Denmark) and Complete Freund’s adjuvant (BD Biosciences, Lyngby, Denmark) (200 µg heat-inactivated mycobacterium tuberculosis (BD Biosciences)) subcutaneously in the inguinal region (50 µL in each side). Mice received an intraperitoneal injection of pertussis toxin (300 ng, Sigma-Aldrich, Søborg, Denmark) on day zero and one post immunization. On day 10, mice were anesthetized with isoflurane inhalation, and using a stereotactic frame, a 30-gauge needle attached to a 50 µL Hamilton syringe was inserted in the right and left hemisphere 1.6 mm deep into the CC (a schematic representation is shown in Figure 1a). Stereotactic coordinates were relative to bregma 1 mm anterior, 1 mm lateral, and 1.6 mm ventral. A total volume of 2 µL PBS was infused at 0.4 µL/min. Mice received Temgesic (RB Pharmaceuticals Limited, Berkshire, UK) for pain relief and isotonic NaCl subcutaneously to prevent dehydration. On day 16, the mice were sacrificed. Mouse genotypes were blinded before immunization and remained blinded until after data analysis. Mice were monitored for weight loss and symptoms associated with EAE. The EAE grades were as follows: Grade 0; no signs of disease; Grade 1 weak tail tonus or hook tail; Grade 2, floppy tail or complete loss of tonus in the tail; Grade 3, floppy tail and hind limb paresis; Grade 4, floppy tail and unilateral hind limb paralysis; Grade 5, floppy tail and bilateral hind limb paralysis. Due to ethical considerations, mice were sacrificed within 24 h of reaching Grade 5.

### 2.3. Propidium Iodide

Thirty minutes before sacrifice, mice were anesthetized by isoflurane inhalation. A 30-gauge needle (bent at 55°, 2–2.5 mm from the tip) attached to a 50 µL Hamilton syringe was inserted between the skull and the cervical vertebra into the intrathecal space of the cisterna magna. A total volume of 10 µL containing 1 µg/µL propidium iodide (Sigma-Aldrich) was injected. Anesthesia and analgesia were as described above.

### 2.4. Tissue Processing

Mice were euthanized through an overdose of sodium pentobarbital and subsequently perfused transcardially with ice cold PBS (20 mL). For histology, brains were post-fixed in 4% paraformaldehyde, immersed in 30% sucrose in PBS at 4 °C, then frozen in cryostat embedding medium (Killik, Milano, Italy) by immersion in 2-methylbutane (Sigma-Aldrich) in liquid nitrogen. Serial coronal sections of 12 µm thickness spanning the lesion were cut on a Micron HM 550 cryostat. For RNA extraction, the CC was micro-dissected and placed in 100 µL RLT buffer (Qiagen Denmark, Kastrup, Denmark). Flow cytometry was carried out on cells isolated from the CC with lesions.

### 2.5. Histology

For examination of the focal EAE pathology, serial sections were stained for MOG and hematoxylin and eosin (H&E).

In brief, 12 µm sections were treated with 0.3% hydrogen peroxide to exhaust endogenous peroxidase activity, then washed in PBS containing 0.2% Triton X-100 (Sigma-Aldrich) and non-specific staining was blocked using 3% bovine serum albumin (Sigma-Aldrich). Sections were incubated with the primary antibodies biotinylated anti-MOG (1:50) (hybridoma clone Z2), then washed and incubated with streptavidin-horseradish peroxide (1:200) (GE Healthcare Biosciences AB, Danderyd, Sweden). Sections were then washed and developed with 0.5 mg/mL 3,3′-diaminobenzidine (Sigma-Aldrich), then dehydrated using increasing concentrations of ethanol, cleared in xylene, and mounted using DPX mounting medium (Merck KGaA, Darmstadt, Germany).

For H&E staining, sections were dried then immersed in cold methanol for 10 min. After washing in running water, sections were immersed in Meyers hematoxylin staining solution (Sigma-Aldrich) for 15 min, then washed in running water before staining in 0.5% eosin (Sygehusapotek Fyn, Odense, Denmark) for 30 s. Sections were then washed and dehydrated, cleared, and mounted as described above.

### 2.6. Flow Cytometry

Isolated CC was dissociated using Multi Tissue Dissociation Kit 1 (Miltenyi Biotec, Bergisch Gladbach, Germany) according to the manufacturer’s protocol. A single cell suspension was generated by forcing samples through a 70-mm cell strainer (BD Biosciences). Myelin was cleared from the samples by centrifugation on a 37% Percoll gradient (GE Healthcare Biosciences AB). Samples were washed in HBSS + 2% fetal bovine serum (FBS) (Sigma-Aldrich). Samples were first incubated with blocking solution, FBS (Sigma-Aldrich), anti-Fc receptor antibody (BD Biosciences), and hamster IgG (Jackson ImmunoResearch, West Grove, PA, USA) in HBSS, then with anti-CD45 (30-F11; Biolegend, San Diego, CA, USA), anti-CD11b (M1/70; Biolegend), anti-TCRbeta (H57-597; Biolegend), anti-CD4 (rm-4-5; BD Biosciences or Biolegend), anti-CD8 (53-6.7; Biolegend), anti-CD11c (HL3; BD Biosciences; Isotype control), and anti-PD-L1 (B7-H1; BioLegend; Isotype control). Data were collected on an LSRII™ flow cytometer (BD Biosciences) or on a FACSARIA III (BD Biosciences) and analyzed using Flowlogic^TM^ 6 (Inivai Technologies). Preparation, data collection, and analysis were conducted on blinded samples.

### 2.7. RNA Sequencing

RNA sequencing (RNA-Seq) was performed on RNA from isolated CC using the RNeasy Micro Kit (50) (Qiagen) according to the manufacturer’s protocol. Previously, RNA-Seq was performed by the Genome Analysis Facility of the Odense University Hospital [20]. In brief, the RNA quality was checked (Agilent bioanalyzer, Santa Clara, CA, USA), sequence libraries were prepared (Illumina Truseq Stranded mRNA Sample Preparation Kit, San Diego, CA, USA), and sequencing was performed (Illumina NextSeq sequencer using 2 × 75 bp paired end reads). FASTQ files were created (CASAVA) then processed (BBDUK from the BBMAP tool package), and low quality bases and reads were removed (k-mer-filtering). Reads were first aligned against the murine transcriptome (Mus_musculus. GRCm38.87), and then against the murine genome (Mus_musculus. GRCm38) using TOPHAT2. The HTseq-count python module was used for quantification. Raw counts were transformed into counts per million (cpm) using the edgeR R-package. Only genes that had cpm ≥ 1 cpm in at least three samples were kept for further analysis. The edgeR package was used for TMM normalization. Finally, statistics for differential expression analysis were calculated with the limma R-package. Heatmaps were generated using the gplot R-package. ISGs were identified using the Interferome database [21]. Genes were selected as being ISGs based on in vivo evidence from mus musculus (fold change (FC) 1.5, *p* < 0.05). KEGG pathway enrichment analysis was performed using DAVID [22,23] with Fisher’s exact test (corrected *p* < 0.05, Bonferroni correction) and then visualized using the ggplot2 R-package.

### 2.8. BioMark, Fluidigm Analysis

Fifty ng RNA isolated from the CC using the RNeasy Micro Kit (50) (Qiagen) was converted to cDNA. Specific targets were pre-amplified in one step by using the CellDirect One-Step qRT-PCR Kit (Invitrogen, Carlsbad, CA, USA) according to the manufacturer’s instructions. Briefly, 50 ng RNA was combined with SUPERase-In (Ambion, Austin, TX, USA), SuperScript II RT/Platinum Taq mix (Invitrogen), and 0.2× of all primers used in the present study (TaqMan gene expression assays, Applied Biosystems, Foster City, CA, USA). PCR with reverse transcription thermal was performed using the thermal cycling protocol: RT (incubated 50 °C for 15 min), Taq activation (95 °C for 2 min), followed by 15 cycles of (95 °C at 15 s and 60 °C at 4 min). Pre-amplified cDNA was diluted at least 1:5 in low EDTA TE-buffer (VWR—Bie & Berntsen, Søborg, Denmark) before real-time qPCR on the BioMarker (Fluidigm, South San Francisco, CA, USA). qPCR was performed using the 48.48 Dynamic Array Integrated Fluidic Circuits (Fluidigm) combining 48 pre-amplified samples with some of the primers as above-mentioned. Reagents used for 48 reactions of pre-sample mix: 3 µL of TaqMan gene expression master mix (Applied Biosystems), 0.3 µL of 20× GE sample loading reagent (Fluidigm), 6 µL low EDTA TE buffer (VWR—Bie & Berntsen), and 2.7 µL diluted pre-amplified cDNA. Primer mixes: 2.5 µL of 20× TaqMan gene expression assays (Applied Biosystems) as described above and 2.5 µL of 2× assay loading reagent (Fluidigm). The 48.48 Dynamic Array was primed in the IFC controller (Fluidigm) before loading the sample and primer. The sample mix, including cDNA (5 µL) and primer mix (5 µmL), was dispensed into appropriate inlets and loaded into the chip in the IFC controller, combining each sample with each primer pair in separate reactions. The plate was placed in the BioMark PCR instrument (Fluidigm), and the standard protocol was followed. Data were acquired using the Fluidigm Real-Time PCR Analysis software 3.0.2 (Fluidigm).

Only data that passed the internal quality control were included in the analysis. Data were first normalized to the housekeeping gene GAPDH using the formula POWER (2, housekeeping gene—gene of interest) where the housekeeping gene was compared to all genes of interest for all samples. Next, fold change (FC) was calculated either relative to C57BL/6 naïve or to the average of the C57BL/6 EAE group, using five of six samples, as one sample had data points that failed the quality control. For the heatmap visualization, a log2 transformation of the fold change values to C57BL/6 EAE was applied. A double gradient colormap was used to create a heatmap in GraphPad Prism 10.

### 2.9. Quantification of Pathology

Images were acquired using an Olympus BX51 microscope with an Olympus DP73 camera (Olympus, Ballerup, Denmark). Lesion pathology was analyzed on serial sections spanning the full lesion in CC for both MOG and H&E staining. Sectioning, staining, imaging, and analysis were conducted on blinded samples.

Demyelination was defined as loss of MOG (myelin) staining and quantified as a percentage of the corresponding CC for each section analyzed. The area of lost staining in CC (Figure 1a, red line) was measured using Fiji, ImageJ version 2.14.0/1.54f. The individual area of loss was then divided by the measured area of the CC (Figure 1a, blue line) in that hemisphere and expressed as a percentage.

$$demyelination=\frac{measured\;loss\;of\;MOG\;staining\;in\;CC}{measured\;area\;of\;CC}\times 100$$

The section with the greatest loss from each mouse was taken as representative of the lesion and used for statistical analysis. Infiltration was identified by H&E staining; quantification was carried out by counting the number of sections with an infiltrate in CC.

### 2.10. Statistics

The D’Agostino–Pearson omnibus K2 normality test was used to test for Gaussian distribution when the n in individual experiments was high enough. We assumed a nonparametric distribution for individual experiments where n was too small for the test. Data were then examined for outliers using the ROUT test (Q = 1), and identified outliers were removed before further testing. Significant differences were identified using the parametric unpaired *t* test or nonparametric Mann–Whitney test. For analysis of EAE incidence, Fisher’s exact test was performed, while the EAE disease score was analyzed using non-parametric unpaired multiple Mann–Whitney tests. Statistical analysis was conducted using GraphPad Prism version 10 (GraphPad Software Inc., San Diego, CA, USA). Values of *p* < 0.05 were considered statistically significant.

## 3. Results

### 3.1. Focal EAE Pathology in Corpus Callosum Is Influenced by Type I IFN Signaling

Female C57BL/6 mice received a standard immunization for EAE. Ten days post-immunization, a focal lesion was induced by inserting a 30 G needle to the CC and injecting 2 uL PBS (Figure 1a). Day 16 post-induction was previously identified as the optimal time point for analysis of the lesions [6]. Histological examination of the lesion confirmed previous observations [6] of local demyelination in CC, identified as a loss of MOG staining, (Figure 1b,c) co-localized with classical EAE infiltrates, and identified by hematoxylin and eosin staining (Figure 1d).

To further our understanding of the development of the focal lesion, we compared transcriptomes of lesioned CC to non-immunized (naïve) CC. Out of the 15,246 genes examined, 710 were identified as differentially expressed (Log2 fold change (LogFC) ≥ ±1.5 and false discovery rate (FDR) ≤ 0.05) (Figure 1e). Of these, 581 genes were upregulated (Figure 1f, top) and 129 were downregulated (Figure 1f bottom). Among the differentially expressed genes, the frequency of type I IFN stimulated genes was 49.6% (288 out of 581) of upregulated and 14% (18 out of 129) of downregulated genes. Though some upregulation of ISGs would be caused by the needle insertion, their role in focal lesion formation still warrants closer examination. KEGG pathway enrichment analysis of the upregulated ISGs identified several enriched pathways including pathways of PRR signaling, cytokine/chemokine signaling, phagocytosis, and apoptosis (Figure 1g). Interestingly, we identified the RIG-I-like receptor signaling pathway, which we previously showed to suppress EAE [5]. Also, the TLR9 signaling and NOD2 signaling pathways were identified, whose activation we previously showed to be beneficial in both conventional EAE and in the focal EAE model [6]. Another significantly enriched pathway identified was the cytosolic DNA sensing pathway. This pathway was of interest as cell-free DNA, identified by intrathecal injection of propidium iodide immediately prior to sacrifice, was observed in the focal lesions (Figure 1h).

### 3.2. STING Deficiency Increased Focal Lesions in Female Mice

A central hub for the cytosolic DNA sensing pathway is STING, a double stranded DNA sensor. Contrasting reports regarding STING’s role in EAE development [17,18,24] have identified this cytoplasmic receptor as an interesting avenue for investigation. To examine the role of STING in focal lesion development, STING-deficient mice (STING^gt/gt^) and C57BL/6 (WT) female mice were immunized, and lesions were directed to CC.

Although this study was not designed to evaluate the EAE score (motor symptoms) nor spinal cord pathology, we observed a significantly higher proportion of STING-deficient mice that remained asymptomatic (*p* = 0.029), with no significant differences in disease onset nor maximum clinical score. The STING-deficient mice did lose significantly more weight at day 16 post-immunization compared with C57BL/6 (*p* = 0.016) (Table 1).

**Figure 2:**
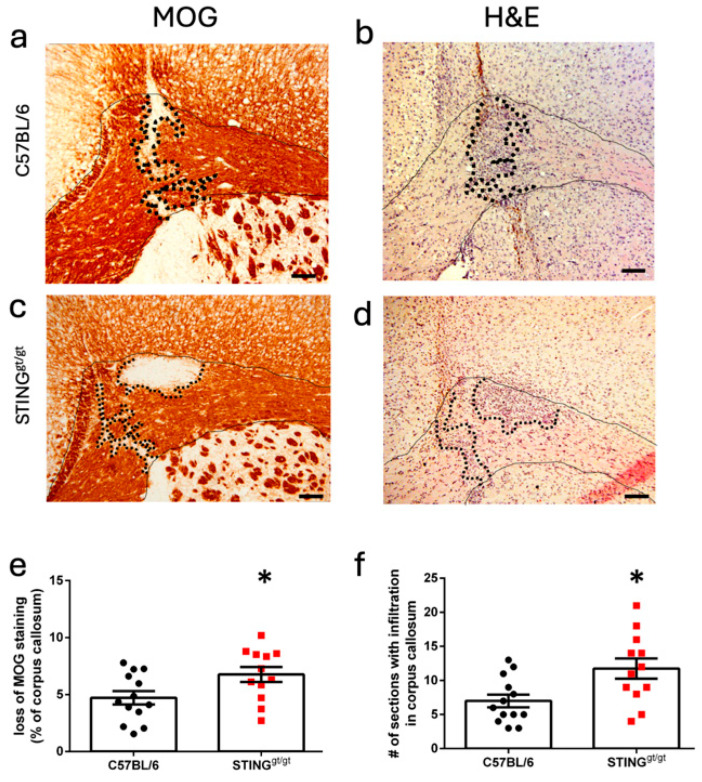
STING-deficient mice developed significantly larger focal lesions in CC. C57BL/6 and STING-deficient (STING^gt/gt^) female mice were immunized with the MOG 35-55 peptide and pertussis toxin. Lesions were localized by stereotactic needle insertion to the corpus callosum at day 10, and mice were sacrificed at 16 days post-immunization. (**a**) Representative micrograph of focal lesion in CC (solid lines) showing demyelination (dotted lines) identified using myelin oligodendrocyte glycoprotein (MOG) immunohistochemistry in a C57BL/6 mouse. (**b**) Representative micrograph of focal lesion in CC (solid lines) showing classic infiltration (dotted line) identified with hematoxylin and eosin (H&E) staining in a C57BL/6 mouse. (**c**) Representative micrograph of focal lesion in CC (solid lines) showing demyelination (dotted lines) identified using MOG immunohistochemistry in a STING-deficient mouse. (**d**) Representative micrograph of focal lesion in CC (solid lines) showing classic infiltration (dotted line) identified with H&E staining in a STING-deficient mouse. (**e**) Bar graph of the quantification of CC demyelination in C57BL/6 vs. STING-deficient mice, represented as a percentage of CC that lost MOG immunohistochemistry staining. (**f**) Bar graph of the quantification of the number of sections showing infiltration in CC in C57BL/6 vs. STING-deficient mice. Bar graphs show pooled results from two separate experiments: *n* = 13 for C57BL/6 and *n* = 12 for STING-deficient mice; C57BL/6 samples are represented as black dots; and STING-deficient samples as red squares. Data are shown as the mean ± SEM. Statistical test: unpaired *t* test. *p* ≤ 0.05 was considered a statistical difference, * ≤ 0.05. Scale bar: 100 µm.

While CC lesions do not affect motor symptoms, we observed a weak correlation between EAE grade and lesion size (Pearson r (R^2^ = 0.33, *p* = 0.041)), confirming previous published results [6]. Importantly CCs in C57BL/6 and in STING-deficient mice were equivalent in gross appearance and size (mean (SD), 45,663 (3151) and 46,881 (5963) pixels, respectively, *p* = 0.52). Both the WT and STING-deficient mice developed CC lesions with demyelination (Figure 2a,c) and infiltration (Figure 2b,d). Quantification of lesion pathology showed that STING-deficient mice developed significantly larger focal lesions compared with the C57BL/6 mice. We observed a significant increase in the percentage of demyelination in CC in STING-deficient mice (Figure 2e) and a significant increase in the number of sections showing a classical EAE-infiltrate in CC in STING-deficient mice (Figure 2f). All mice analyzed for pathology (C57BL/6 *n* = 13, STING *n* = 12) showed lesions in CC, independent of clinical EAE status.

### 3.3. Lowered ISG Expression Was Associated with Focal Lesions in STING-Deficient Mice

**Figure 3:**
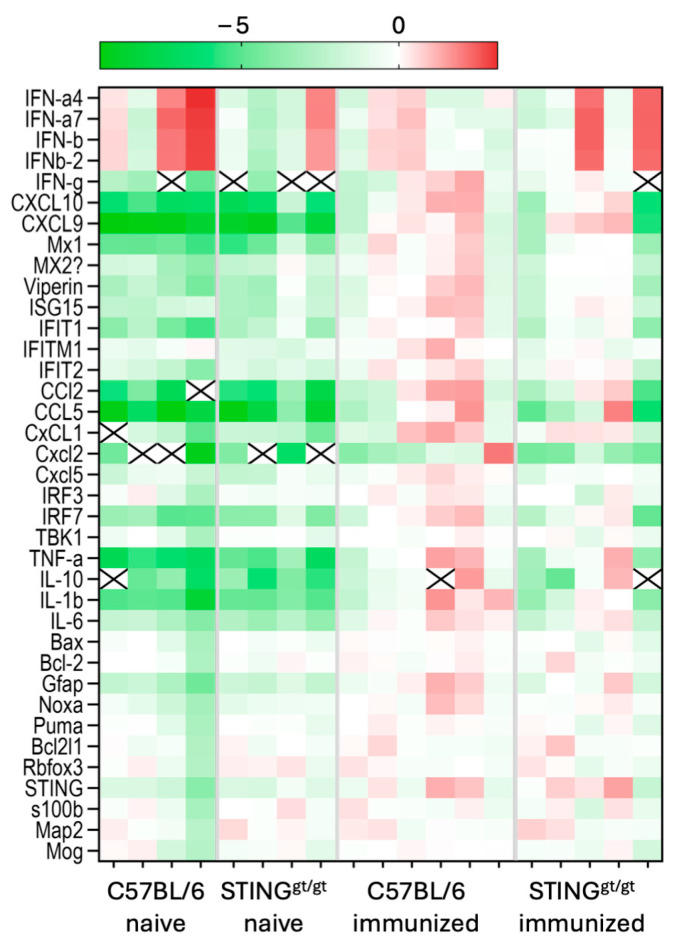
Modified ISG levels were associated with focal lesions in STING-deficient mice. C57BL/6 and STING-deficient (STING^gt/gt^) female mice were immunized with the MOG 35-55 peptide and pertussis toxin. Lesions were localized by stereotactic needle insertion to the corpus callosum on day 10 and CC was micro-dissected at 16 days post-immunization. Micro-dissected CC from unmanipulated (naïve) C57BL/6 and STING-deficient mice was also collected. RNA was extracted and several selected ISGs were analyzed by the BioMark, Fluidigm multiplex array. A heatmap generated from FC calculated to the C57BL/6 immunized samples with red representing increased expression and green representing decreased expression compared with the C57BL/6 immunized samples. X indicate sample was below detection limit for this gene.

STING activation induces IFN I production, which in turn promotes the expression of ISGs involved in immune regulation. Multiple ISGs were examined from lesioned and non-immunized (naïve) CC from both C57BL/6 and STING-deficient mice using the BioMark, Fluidigm multiplex assay. Fold changes (FC) was calculated to lesioned CC from C57BL/6 (C57BL/6 immunized) to better access changes between the two lesioned groups. A heatmap showed the decreased expression of several of the ISGs examined in lesioned CC from STING-deficient mice compared with C57BL/6 (Figure 3). Both lesioned groups showed increased expression compared with naïve CC from STING-deficient and C57BL/6 mice.

### 3.4. STING/Deficient Mice Show Altered Infiltration

**Figure 4:**
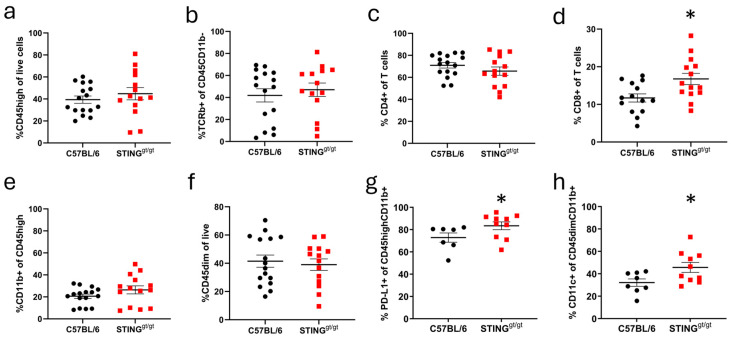
Altered immune infiltration in lesions form STING-deficient mice. C57BL/6 and STING-deficient (STING^gt/gt^) female mice were immunized with the MOG 35-55 peptide and pertussis toxin. Lesions were localized by stereotactic needle insertion to the corpus callosum on day 10 and CC was micro-dissected at 16 days post-immunization for flow cytometry analysis. (**a**) Percentages of CD45high cells (infiltration from peripheral blood) in the live gate. (**b**) Percentage of TCRb+CD45highCD11b- cells (T cells) in the CD45high population. (**c**) Percentage of CD4+ cells in the TCRb+CD45highCD11b- population. (**d**) Percentage of CD8+ cells in the TCRb+CD45highCD11b- population. (**e**) Percentages of CD11b+ cells (monocytes/macrophages) in the CD45high population. (**f**) Percentage of CD45dimCD11b+ cells (microglia) in the live gate. (**g**) Percentages of PD-L1+ cells in the CD45highCD11b+ population. (**h**) Percentages of CD11c+ cells in the CD45dimCD11b+ population. (**a**–**f**) Scatter plots show pooled results from three separate experiments, *n* = 16 for C57BL/6 and n = 14 for STING-deficient mice, (**d**) two outliers were removed from the C57BL/6 group. (**g** + **h**) Scatter plots show one experiment, *n* = 9 for C57BL/6 and *n* = 10 for STING-deficient mice; for analysis of this dataset, two outliers were removed from macrophages in C57BL/6 and one from microglia in C57BL/6 mice. C57BL/6 samples are represented as black dots and STING-deficient samples as red squares. Data are shown as the mean ± SEM. Statistical test: unpaired *t* test but for (**g**) Mann–Whitney test. *p* ≤ 0.05 was considered a statistical difference, * ≤ 0.05.

Immune infiltration was analyzed through the flow cytometry of micro-dissected lesioned CC. There was no difference in total infiltration between C57BL/6 and STING-deficient mice (CD45high, Figure 4a). Further analysis of the major infiltrating populations revealed no overall difference in the T cell population (CD45highCD11b-TCRb+, Figure 4b), and while CD4+ T cells were the predominant population for both C57BL/6 and STING-deficient mice (Figure 4c), STING-deficient mice showed a significant increase in the proportion of CD8+ T cells (Figure 4d). No differences were observed in the proportions of infiltrating monocytes/macrophages (CD45highCD11b+, Figure 4e) nor resident microglia (Figure 4f). However, we observed changes in the myeloid cell populations, whereby the monocytes/macrophages exhibited a significantly higher PD-L1 expression (Figure 4g) in STING-deficient mice, indicating an immunoregulatory phenotype, and microglia in STING-deficient mice showed increased CD11c expression (Figure 4h), suggesting an altered activation state compared with C57BL/6.

## 4. Discussion

This study provides critical insights into the regulatory role of STING in the development of neuroinflammatory and demyelinating lesions. Our findings revealed that STING deficiency exacerbates lesion size and alters ISG expression levels and immune cell infiltration dynamics. These observations suggest that STING serves as a key modulator of both innate and adaptive immune responses in the CNS, with potential therapeutic implications for MS and other demyelinating disorders.

This study utilized a newer focal EAE model [6] that resulted in the formation of demyelinating lesions in CC, which do not occur in conventional EAE models. The model is an extension of the existing MOG35-55 EAE in C57BL/6 mice and has many of the same limitations (e.g., being based on adjuvants used in immunization). However, we found that the focal model was more reliable, as all mice analyzed for pathology developed a well-defined, temporally staged lesion in a targeted location whereas classical EAE developed defuse, temporally undefined lesions. This makes the focal EAE model highly desirable when investigating the mechanism behind lesion development and resolution as it allows for precise comparison between groups. In the focal EAE model, lesion development occurred independent of the development of clinical signs, though, as previously published [6], there was a weak correlation in C57BL/6 mice between lesion size and EAE clinical severity. As the lesion is directed using a stereotactic injection, mechanical damage cannot be avoided, but the needle was not inserted to the CC itself. Previously published non-immunized controls excluded that mechanical damage in the cortex directly influenced the CC lesions. The model results in a focal lesion, making it ideal for the evaluation of treatment effects in lesion development and repair, as we have previously shown [6]. While existing controls from previous publications provide reassurance regarding the specificity of lesion induction, the absence of a sham group in the current experimental setup remains a notable limitation and should be considered when interpreting lesion-specific effects.

Transcriptomic analysis demonstrated that ISGs constitute a significant proportion of the upregulated genes in lesions, and the KEGG pathway enrichment analysis indicated several PPR pathways such as Toll-like receptors and NOD-like receptors, which are important in regulating inflammatory cascades in the CNS. While Toll-like receptors (TLRs) have been extensively studied in the context of EAE, their role in disease pathogenesis remains somewhat controversial, with studies showing both pathogenic and protective effects depending on the specific TLR and disease stage involved [25,26,27,28]. This complexity has made it difficult to draw definitive conclusions about their function in EAE. In contrast, our findings support a more consistent and specific role for the STING pathway in modulating neuroinflammation. The divergence in the immunological outcomes associated with TLR versus STING signaling highlights the importance of further dissecting innate immune pathways in CNS autoimmunity. As a critical component of the innate immune system, STING functions as a PRR in the cytosolic DNA sensing pathway, a pathway that was also significantly enriched in the KEGG pathway analysis. This pathway is essential for detecting cytosolic DNA released from pathogens and damaged cells, resulting in an amplified type I IFN response [2], and promotes localized immune activation [24]. Detection of cell-free DNA within lesions, as indicated by propidium iodide staining, supports that STING-mediated cytosolic DNA sensing is crucial for initiating protective type I IFN signaling and constraining lesion expansion [24,29]. Previous studies have reported conflicting roles for STING in EAE. Some suggest that STING activation might exacerbate disease severity, while others show that STING activation can suppress disease severity through type I interferon responses [16,17,18]. Our findings demonstrate that STING deficiency leads to enhanced demyelination and immune cell infiltration in focal EAE lesions, supporting a beneficial role for STING activation. The discrepancy in the literature may be due to differences in disease models, the timing of STING activation, or the balance between protective and pro-inflammatory pathways downstream of STING signaling. By focusing on focal lesions, our study provides new insights into the spatial and functional dynamics of STING in CNS autoimmunity.

The significantly larger demyelinated lesions in STING-deficient mice support this protective role of STING in limiting lesion expansion during neuroinflammation. This aligns with previous findings indicating that STING activation mitigates tissue damage by modulating neuroinflammatory responses [17,18,24]. The analysis of ISGs demonstrated a marked downregulation in STING-deficient lesions. STING activation is a well-established driver of type I IFN production, which in turn induces ISG expression involved in immune cell recruitment, activation, and the resolution of inflammation [16,24]. The downregulation reinforces the hypothesis that STING signaling mediates protection, as ISGs are crucial modulators of neuroinflammation, acting to limit excessive immune responses and protect CNS integrity. The downregulation may impair these protective mechanisms, leading to heightened inflammation and lesion expansion, further substantiating the importance of STING in modulating neuroinflammatory diseases [16].

The increased lesion size observed in the absence of STING likely results from dysregulated immune responses, characterized by excessive inflammatory cell infiltration and sustained tissue damage. Histological analyses revealed an increase in cellular infiltration within STING-deficient lesions. Notably, flow cytometry analysis showed an enrichment of CD8+ T cells. CD8+ T cells are well-documented mediators of neurotoxicity, contributing to axonal injury and demyelination in MS and EAE models [30]. These cytotoxic lymphocytes can trigger oligodendrocyte and neuronal apoptosis, thereby exacerbating CNS pathology. The observed increase in CD8+ T cells in STING-deficient mice suggests a failure to regulate T cell activation and CNS migration, fostering a more aggressive inflammatory milieu. This supports the hypothesis that STING plays a crucial role in maintaining the balance between pro-inflammatory and anti-inflammatory immune responses during neuroinflammation [24]. Future investigations should aim to elucidate the mechanistic link between STING activation and CD8+ T cell infiltration as well as the long-term effects of STING-targeted therapies on CNS inflammation and neurodegeneration. However, further functional characterization will be necessary in future work to definitively establish their effector or regulatory roles.

STING is known to activate downstream signaling pathways including TBK1 [16], leading to type I interferon production, and may also modulate NF-κB-dependent pro-inflammatory responses, which could influence microglia activation, cytokine release, and the recruitment of peripheral immune cells, making them potential targets for the therapeutic modulation of STING-mediated neuroinflammation [31].

Although the proportions of infiltrating macrophages and resident microglia did not significantly differ between STING-deficient and WT mice, their activation states were markedly distinct. Macrophages in STING-deficient mice exhibited significantly higher PD-L1 expression, indicative of an immunoregulatory phenotype. PD-L1 is known to suppress excessive immune activation and prevent tissue damage [32], suggesting a potential compensatory mechanism in response to heightened inflammation. Likewise, microglia from STING-deficient mice displayed increased CD11c expression, a marker associated with altered activation states that may influence myelination, cellular infiltration, and cytokine production [33]. These findings underscore STING’s role in modulating myeloid cells and microglia activation during neuroinflammatory conditions.

## 5. Conclusions

Overall, our study delineates STING’s critical role in shaping lesion pathology, immune infiltration, and ISG expression in focal EAE. The increased lesion size and altered immune landscape in STING-deficient mice indicate that STING signaling constrains local neuroinflammation and modulates immune responses within demyelinating lesions. These findings position STING as a promising therapeutic target for modulating neuroinflammation in MS and related disorders.

## Figures and Tables

**Table 1:** STING-deficient mice had a lower incidence in EAE. Comparison of incidence, time of onset, maximum severity, and weight change (%) at day 16 post-immunization between STING-deficient and C57BL/6 mice.

	C57BL/6	STING^gt/gt^	*p*-Value
Incidence	24/44	12/40	0.029 Fisher’s exact test
Time of onset	13.83 ± 0.31	13.25 ± 0.35	0.216 Mann-Whitney test
Maximum severity	2.71 ± 0.28	3.33 ± 0.36	0.174 Mann-Whitney test
Weight change at day 16 post immunization (%)	104.8 ± 7.1	100.7 ± 8.4	0.016 Mann-Whitney test

## Data Availability

The raw data supporting the conclusions of this article will be made available by the authors on request.

## Abbreviations

The following abbreviations are used in this manuscript:

PPR	Pattern recognition receptor
IFN	Interferon
EAE	Experimental autoimmune encephalomyelitis
MS	Multiple sclerosis
STING	stimulator of IFN genes
CNS	Central nervous system
ISGs	Interferon stimulated genes
TLR	Toll-like receptor
WT	Wild type
CC	Corpus callosum
MOG	Myelin oligodendrocyte glycoprotein
µl	Microliter
H&E	Hematoxylin and eosin
FC	Fold change
